# The myth of 2.5 cm symphyseal diastasis

**DOI:** 10.1007/s00402-025-05904-x

**Published:** 2025-05-21

**Authors:** Axel Gänsslen, Jan Lindahl, Dietmar Krappinger, Richard A. Lindtner, Mario Staresinic

**Affiliations:** 1 Hannover Medical School, Hanover, Germany; 2 Johannes Wesling Klinikum Minden, Minden, Germany; 3 Helsinki University Hospital, Helsinki, Finland; 4 Innsbruck Medical University, Innsbruck, Austria; 5 Zagreb University Hospital “Merkur” Zagreb, Zagreb, Croatia

**Keywords:** Pubic symphysis, Symphyseal width, Symphyseal physiology, Instability, Consequences

## Abstract

Detection of disruption of the pubic symphysis and resulting anterior pelvic ring instability primarily depends on the symphyseal widening on standard anterior–posterior X-rays. Based on biomechanical and clinical analyses from the 80 to 90’s, a cut-off value of 2.5 cm widening distinguished between stable and unstable lesions. A relevant debate developed concerning minor (< 2.5 cm displacement), moderate (> 2.5 cm displacement) and severe disruptions (> 2.5 cm displacement + posterior complete pelvic ring instability) of the pubic symphysis. Analysis of anatomic, biomechanical, physiological and clinical literature showed, that an exact value does not allow this differentiation. Thus, symphyseal posttraumatic disruptions with displacements > 10 mm should be treated surgically, while in minor displacements (5–10 mm) stress examination can guide adequate treatment.

## Introduction

Traumatic disruption of the pubic symphysis usually occurs after an anteroposterior compression (APC) mechanism due to external rotation of one or both hemipelves, which additionally results in a variable degree of injury to the SI-joint(s), either partially or complete.

Disruption of the pubic symphysis has to be analyzed as part of a pelvic ring injury and therefore treatment considerations should always additionally consider the posterior pelvic ring injury.

Open reduction and internal fixation (ORIF) is the standard of care in symphyseal disruptions [1]. In contrast, external fixation is limited to cases that need emergency stabilization [2] as its use as a definitive treatment results in long healing times [3] and unacceptable outcomes [4].

The present gold standard in treating symphyseal disruption is symphyseal plating [5–12] with at least two screws on each side [1] based on excellent biomechanical results [13–15], despite new developments also show encouraging results, including endoscopic-assisted stabilization methods [16], irrespective of existing posterior pelvic ring injury.

Symphyseal ruptures were probably the first surgical interventions performed on the pelvic ring. Already in 1911, Finsterer treated an older symphysis rupture by using an aluminum-bronze wire [17], and Lambotte in 1913 described a dynamic technique using a wire cerclage or a screw osteosynthesis [18].

Classical indications for plate stabilization of the pubic symphysis include several type B and type C injuries of the pelvic ring, where the symphyseal diastasis is part of a Tile type B or C injury of the pelvic ring.

A posttraumatic symphyseal width of > 15–25 mm was considered an indication for immediate stabilization [19, 20]. Historical recommendations indicate surgery if > 2.5 cm diastasis is present as this corresponds to a clinical “instability” [3].

Based on the instability concept, the 2.5 cm cut-off was integrated into mechanism and stability-based classification systems, leading to surgical treatment decisions, when displacement was > 2.5 cm.

A recent systematic review revealed a clinical trend to a combined anterior and posterior stabilization concept in APC-II injuries, despite heterogenous biomechanical data supporting such a concept. In favor for combined fixation, a significant lower complication rate was reported [21].

In a survey in UK and Ireland, single anterior symphyseal plating and a concomitant single sacroiliac joint (SIJ) screw were the most preferred stabilization methods [22].

## Symphyseal disruptions within osteoligamentous pelvic ring classifications

The most frequently used osteoligamentous classifications are the Young-Burgess classification, the Tile classification, and the AO/OTA classification, the latter is based on the Tile classification.

Tile developed an instability-based classification [23], which focusses on the involvement and integrity of the posterior pelvic ring structures.

In type B1 injuries, e.g. open book injuries as a result of an external rotation mechanism, most often symphyseal disruption is observed. Tile already described three stages, depending on the transferred force to the pelvis:

- Type B1.1: symphyseal separation < 2.5 cm on pelvic a.p. X-ray, no posterior pelvic ring lesion
- Type B1.2: symphyseal separation > 2.5 cm on pelvic a.p. X-ray; unilateral disruption of the anterior sacroiliac ligament and a suspected injury of the sacrospinous ligament
- Type B1.3: symphyseal separation > 2.5 cm on pelvic a.p. X-ray; bilateral posterior injury with disruption of the anterior sacroiliac ligaments and suspected injury of both sacrospinous ligaments

The pelvic ring classification of Young and Burgess additionally distinguishes three stages of this injury based on the displacement of the pubic symphysis [19, 24]:

- APC-I = pubic symphysis diastasis < 2.5 cm without SI-joint ligament injury
- APC-II = symphyseal diastasis > 2.5 cm with anterior SI-joint ligament disruption (= partial posterior injury corresponding to Tile type B injuries)
- APC-III = vertically unstable injury with complete posterior disruption of the SI-joint complex (Tile type C injury)

**Table:** 

Based on these investigations, a cut off value of 2.5 cm symphyseal separation was defined in terms of classification and treatment decisions	

Historically, APC-I injuries were considered stable injuries, thus, non-operative treatment could be favored, while in APC-II injuries, partial posterior injury of the SI-joint complex was observed and isolated anterior (plate) stabilization was considered adequate, as this closes the remaining hinge stability. In contrast, in APC-III injuries with complete posterior ring disruption anterior and posterior stabilization is recommended.

In a FE analysis, APC II injury according to Young-Burgess was simulated in a double leg stance model [25]. The pubic symphysis ligaments showed no relevant loading. Symphysial transection resulted in 1 cm widening when > 800 N force was applied. The pelvic floor ligaments and the interosseous iliosacral ligaments remained intact in this model. Overall, an APC II injury shows horizontal, but no vertical instability. Thus, APC II injuries more correspond to Tile type B injuries as to Tile type C injuries.

During recent years, a grey-zone between APC-I and APC-II injuries was observed, as some APC-I injuries showed higher instability. Additionally, according to biomechanical understanding treatment options were discussed with a trend to additional posterior stabilization in even APC-II injuries.

This analysis deals with the present scientific background on APC-injuries.

## Physiological symphyseal width and asymmetry

The symphyseal space is radiographically often parallel, especially in its posterior part, while an anterior, superior and inferior divergation can be observed.

Especially in women, some asymmetry can be present. The articular hyaline cartilage has a thickness between 0.5 and 3 mm [26–31], which decreases with age [30].

**Fig. 1:**
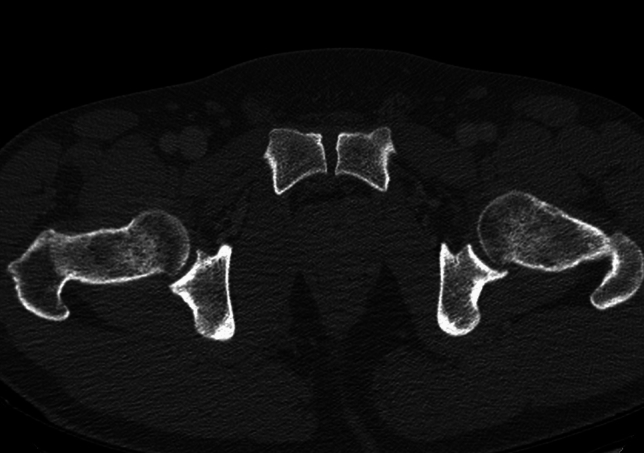
Axial CT view of a pelvis with an uninjured pubic symphysis with anterior physiological widening compared to the posterior symphyseal part

Consensus exists, that the anterior part of the pubic symphysis is wider than posterior part [29, 32–34] (Fig. 1). This should be considered in CT-analyses, as it is unknown were to measure the symphyseal width especially in the axial or Inlet view.

The symphyseal width follows an age- and sex dependent course. Kraus already in 1930 reported, that the width decreases continuously from approximately 10 mm in the toddler age to 2 mm in the older age of 50 years [35]. Patel and Chapman observed a width of 7.4 mm in the first months of life, which decreases to 5.4 mm at the age of 16 [36].

During the last decade, several CT-investigations analyzed the symphysis width in patients of different age:

- Alicioglu et al., performed a CT-analysis on adult patients > 16 years of age in 278 women and 264 men [37]. They reported a constant narrowing of the anterior and posterior part of the symphyseal width, while the middle part showed no significant changes, independent from the number of birth and the body-mass index. Female patients were associated with larger values anterior and in the middle.
- McAlister et al. measured the symphyseal width using standard radiographs in 316 consecutive pediatric patients (165 boys, 151 girls), which were separated by gender and divided into three age groups: 2–6, 7–10, and 11–14 years [38]. In the entire study population normal values between 5.2 and 8.4 mm were observed with an average width of 6.8 mm. Interestingly, a subsequent widening was observed within the three age groups: 6.6, 6.8, 7.2 mm, respectively. It was concluded, that a width > 8.4 mm should lead for further evaluation for pathology.
- Bayer et al. analyzed 350 CT scans of children in different age groups: 0–6, 7–11, 12–15 and 16–17 years. The mean width in these age groups was 5.4, 5.3, 4.1 and 3.5 mm in girls and, 5.9, 5.4, 5.2 and 4.0 mm in boys, respectively [39].
- A further CT-analysis of 1020 CT axial scans in pediatric patients (2–18 years) revealed an average pubic symphyseal width at 2 years old boys of 6.35 mm and of 5.85 mm in girls. A decrease to 3.68 mm in boys and 3.92 mm in girls at an age of 18 years was observed [40].
- Recently, data on 811 CT measurements of the pubic symphysis were analyzed, stating, that from age 2 to 16 years, the average pubic symphysis width decreased from 5.55 to 3.69 m [41].

The width of the pubic symphysis decreases from 5–6 mm two years after birth to 3–4 mm in early adulthood. In pediatric trauma patients < 10 years of age, a symphyseal width > 10 mm should lead to suspicion of injury [40]. Additionally, it is well known, that symphyseal widening occurs under labor [42].

**Table:** 

Normal AP radiographs of the pelvis have a mean symphyseal clear space of 4.4 mm [43]	

## Biomechanics of the pubic symphysis

The basic understanding of pelvic ring trauma biomechanics is based on the extended work of Marvin Tile’s group. Their main conclusions were that “the stability of the pelvic ring depends upon the integrity of the posterior weight-bearing sacroiliac complex, with the major sacroiliac, sacrotuberous and sacrospinous ligaments” [23].

Thus, the posterior pelvis is considered the key element to provide structural support with its stabilizing structures, whereas the anterior ring with the pubic symphysis contributes only little to the intrinsic stability of the ring structure [23].

According to the “key-stone” concept, the posterior ligamentous support is comparable to a suspension bridge [15, 44]. In contrast, the anterior pelvic ring with the pubic symphysis and the pubic arch acts as a pull bar (strut) to prevent lateral spreading [15, 44].

Several authors investigated the effect on pelvic ring instability after sequential dissection of pelvic ligaments to produce an anterior external rotation deformity of the pelvis.

Fundamental studies were first performed by Marvin Tile’s group using a step-by-step instability model [15, 44–46]. Isolated transection of the pubic symphysis led to a maximum symphyseal diastasis of 2.5 cm, while the remaining pelvic floor ligaments and the iliosacral ligaments compensated further rotational and translational movements.

**Table:** 

Again, a cut off value of 2.5 cm symphyseal displacement was confirmed in these biomechanical investigations	

This dissection of the symphysis leads to an approximate reduction of overall pelvic ring stiffness of 40% [23].

Additional transection of the sacrospinous and sacrotuberous ligaments markedly increased “instability”. Only the anterior SI-joint ligaments provided some remaining resistance against rotational (horizontally directed) forces.

Transection of the anterior SI-joint ligaments resulted in complete rotation of the hemipelvis around a “hinge” through the posterior sacroiliac ligaments. Translational movements were prevented by the remaining intraosseous and posterior iliosacral and iliolumbar ligaments, while complete transection of the sacroiliac joint ligament complex made translational movements possible [15, 44–46]. These results were confirmed by Krueger et al. [47].

**Table:** 

This analysis led to the decision, that widening of the pubic symphysis on the pelvic a.p. X ray of ≤ 2.5 cm is considered a stable injury	

In contrast, Dolati et al. analyzed progressive external rotation movements resulting in up to 18 cm symphysial diastasis. Even symphysial widening of 3–4 cm did not result in injury to the SI-joint ligaments, while symphysial widening of 5, 8 and 13 cm resulted in anterior SI-joint ligament injury, anterior and interosseous SI-joint ligament injury with a possible rotational instability and anterior and interosseous SI-joint ligament injury, respectively [48].

Simonian et al. observed a 1.2 mm symphysial gap, no SI-joint displacement and no change of SI-joint angulation (0.43°) after dissection of the symphysial ligaments in uninjured fresh-frozen non-embalmed cadavers [49]. Abdelfattah et al. performed fluoroscopy-based instability testing by manual external rotational force application on six cadaver pelves [50] and reported a mean horizontal displacement of 11.8 mm and a mean vertical displacement of 6.3 mm after dissection of the symphysial ligaments, while the pelvic floor ligaments had no relevant influence on further instability.

Dissection of the SI-joint ligaments resulted in a significant increase of displacement to 38.4 mm and 20 mm, respectively.

**Table:** 

Isolated dissection of the pubic ligaments in uninjured pelves lead to instability of the pubic symphysis of approximately only 1 cm	

Doro et al. showed that anterior sacroiliac disruption is unlikely if symphyseal separation is < 1.8 cm in a static situation and becomes likely, if this separation exceeds 4.5 cm, resulting in grey zone between Young-Burgess’ APC-I (< 2.5 cm displacement) and APC-II (> 2.5 cm displacement) injury groups [51]. In an MRI-based analysis, APC-II injuries were not associated with sacrospinous ligament injury in 50% [52].

**Table:** 

From clinical perspective, it remains questionable, if isolated injury to the symphyseal ligaments without anterior SI joint ligament or pelvic floor ligament injury really exist	

## The 1st grey zone

**Fig. 2:**
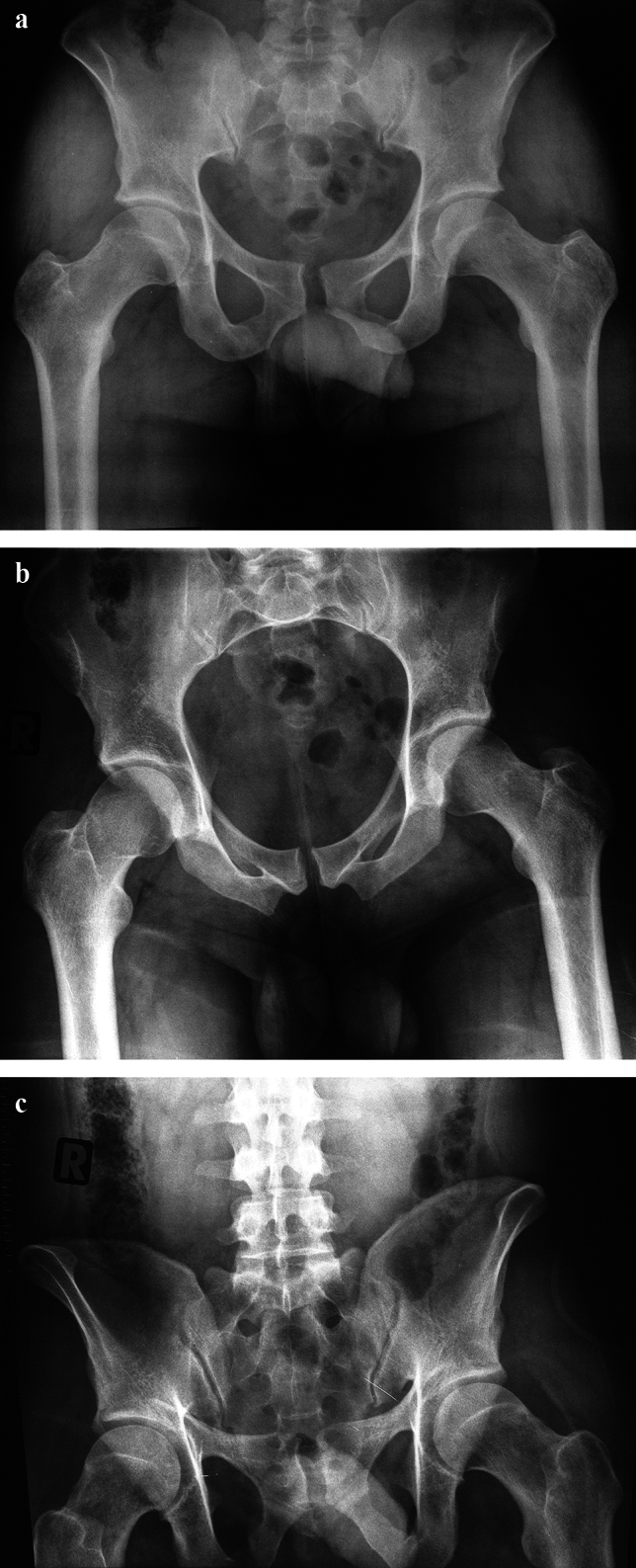
Anterior–posterior compression (APC) injury with small diastasis of the pubic symphysis (approximately 1 cm) in all three standard views (ap. View (**a**), Inlet-view (**b**) and Outlet view (**c**)). Potential involvement of the left SI-joint with slight anterior widening

Based on the physiological separation of both pubic bones at the level of the pubic symphysis, minor injuries (5–10 mm) can result in interpretational difficulties (Fig. 2).

As mentioned, posttraumatic diastasis < 1 cm is extremely rare as in the majority of cases at least anterior ligament injury of an SI-joint is visible on standard conventional X-rays (a.p. pelvis, Inlet, Outlet views) (Fig. 2).

Injury to the pelvic floor ligaments, e.g. the sacrotuberous and sacrospinal ligaments, cannot be visualized on conventional radiographs. Only bony avulsion injuries of these ligaments, most often seen in type C injuries, at the lateral and distal sacrum indicate such injuries, while intraligamentous ruptures can only be visualized with MRI [52] and are therefore undiagnosed in the initial emergency treatment phase.

Under physiological conditions, in single-leg-stance, beside compressive forces, 1.7° internal, 0.8° anterior [53] and caudal rotation [54] of the hemipelvis was observed, resulting in vertical and sagittal shear forces/tension [53, 55] and compression forces acting on the pubic symphysis [53, 54]. In the double-leg-stance especially tensile and distraction forces with symphyseal widening were predominantly observed [14, 53, 54], while sitting leads to tensile forces resulting in a divergence of the pubic rami up to 0.5 mm and an external rotation of the hemipelves of approximately 0.5° [53].

Normal mobility of the pubic symphysis during loading resulted in mobility of up to 2.6 mm in the vertical direction and 1.3 mm in the sagittal direction; comparable mobility was reported during walking with 2.2 and 1.3 mm, respectively [56].

**Table:** 

Symphyseal width < 5 mm and incongruency of 2 3 mm may be considered physiological, without any pathological value	

**Fig. 3:**
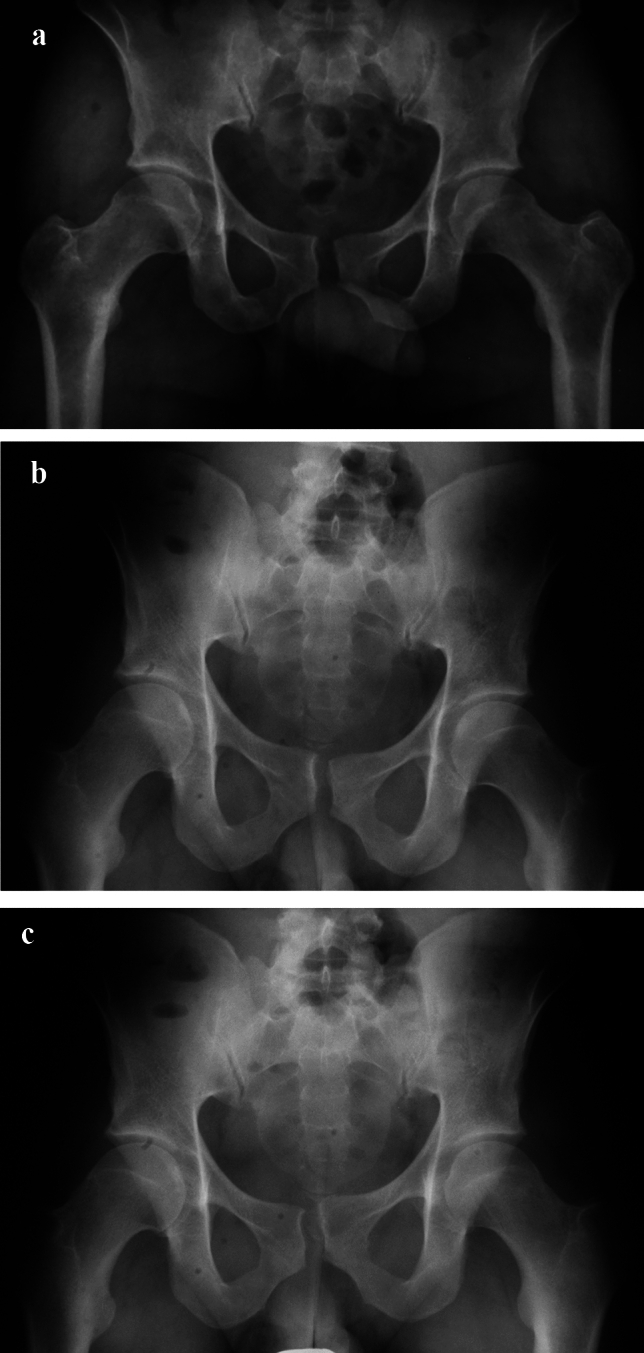
Anterior–posterior pelvic view showing parallel symphyseal widening (**a**). Flamingo views with left (**b**) and right (**c**) weight bearing show some minor vertical displacement

In inconspicuous radiographic analysis of the symphysis, magnetic resonance tomography or bilateral single-leg-stance (flamingo) radiographs can be considered. The latter can show some “step-up” of the pubic bone (Fig. 3).

Single leg-stance Flamingo views show physiologic movement of < 0.5 mm in men and < 1.5 mm in women [57]. In multiparous women ≤ 3 mm width is still normal [57, 58]. Recent analyses of single-leg-stance X-rays [58, 59] showed an average translation of 1.4 mm in males, nulliparous women had an average translation of 1.6 mm and multiparous women an average translation of 3.1 mm [58]. Based on these analyses, 5 mm mobility may act as a cut-off value [59].

**Fig. 4:**
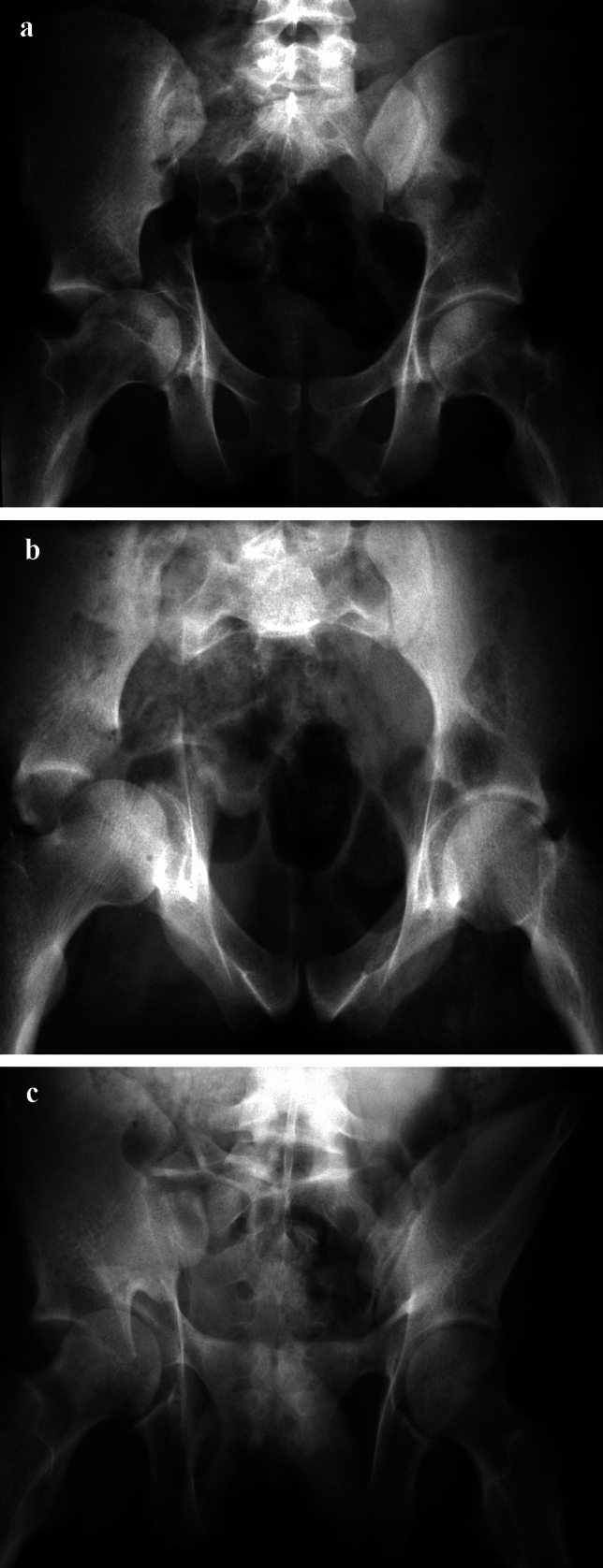
Patient with a typical displacement of a pure right transverse acetabular fracture with classical internal rotation of the obturator segment around a vertical axis through the pubic symphysis (**a**). The Inlet view shows some anterior symphyseal widening compared to the posterior symphyseal axial surface while the Outlet view presents with anatomic symphyseal position (**c**), corresponding to symphyseal strain or to a normal anatomy (see. Figure 1)

In particular, especially acetabular fractures of the transverse family may present with internal rotation of the obturator segment and sometimes external rotation of the iliac segment which may lead to asymmetry and/or some (anterior) widening of the symphysis (Fig. 4). It should be noted, that in these injuries, the symphysis is rarely completely injured.

**Table:** 

Injuries with a maximum of 10 mm symphyseal widening or asymmetry correspond most often to symphyseal strain	

**Fig. 5:**
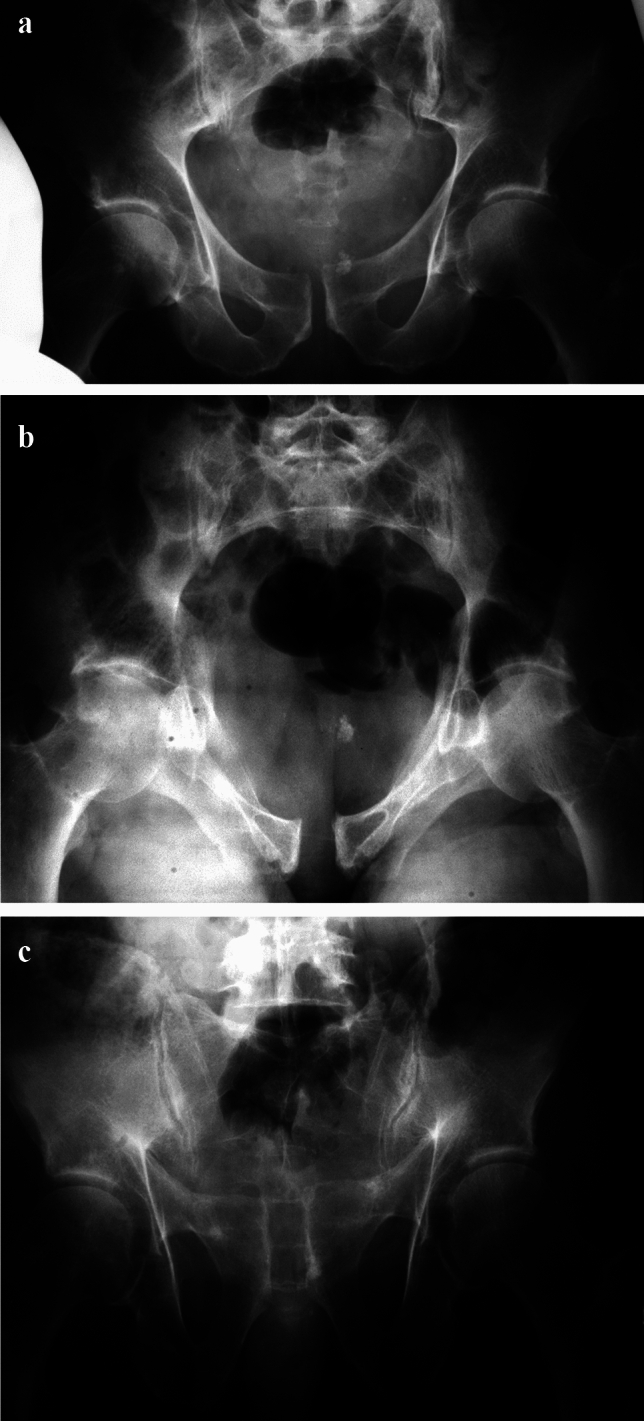
Minor displacement with vertical incongruity of the pubic symphysis after a frontal motor vehicle collision (**a**). Already, Inlet- (**b**) and Outlet-views (**c**) show larger symphyseal displacement with suspected injury to the right SI-joint

It has to be considered, that visualization of 1 cm displacement represents a static situation and may mask true displacement due to reduction forces from muscle power and pain (Fig. 5).

This is well-known after pelvic binder application as application can result in adequate/near anatomic reduction of a displaced pubic symphysis injury. Pelvic binders are recommended during the prehospital phase and are frequently used by prehospital personal [60–63], potentially masking the true amount of symphyseal displacement and therefore instability [64].

## The 2nd grey zone—the 2.5 cm problem

**Fig. 6:**
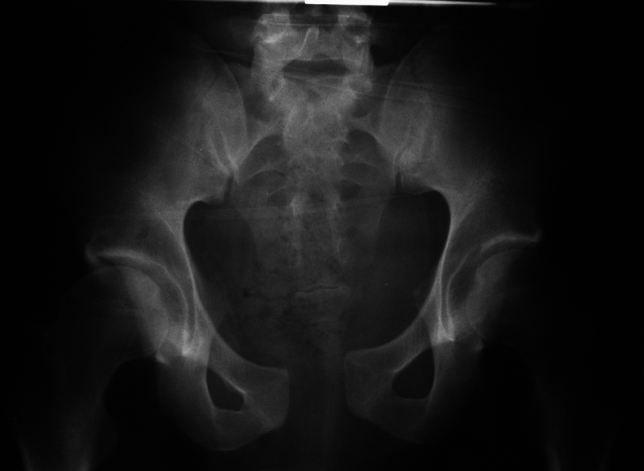
Borderline view of a symphyseal separation of 2–2.5 cm. Based on the existing literature, it remains unclear if this corresponds to instability

Based on the historical data, a symphyseal width of < 2.5 cm was considered a stable injury (Fig. 6). Symphyseal disruption of > 2.5 cm was defined with the presence of anterior sacroiliac ligament injury [23, 24]. Thus, the question arises, is a symphyseal width of 10–25 mm a stable injury?

Recently, Wakefield et al. presented a case of a radiographically APC-I injury who developed on-going symptoms, requiring symphysiodesis [65].

Dolati et al. could show, that symphyseal widening of 3–4 cm did not result in injury to the SI-joint ligaments, while symphysial widening of > 5 cm did [48]. Abdelfattah et al. could show, that only the severing of SI-joint ligaments leads to a further symphyseal diastasis to an average of 38.4 mm horizontal and 20 mm vertical displacement, respectively [50].

Doro et al. showed that anterior sacroiliac disruption is unlikely if symphyseal separation is < 1.8 cm in a static situation and becomes likely, if this separation exceeds 4.5 cm [51]. In this cadaveric model, average symphyseal diastasis was 2.2 cm until anterior sacroiliac ligament disruption was observed (range, 1–4.5 cm), with a gender discrepancy (2.5 cm in males, 1.8 cm in females).

**Table:** 

Experimental set up reveals, that the well known cut off value of 2.5 cm is associated with symphyseal disruption, but not necessarily with ligamentous anterior SI joint injury	

APC-II injuries frequently lead to at least partial failure of the anterior sacroiliac ligament resulting in horizontal instability [25].

Additionally, APC-II injuries are usually not associated with sacrospinous ligament injury [25, 51, 52] which play a role in vertical stabilization [25].

Injury to the interosseous sacroiliac and sacrotuberous ligaments serve as an indicator of vertical instability [25]. The sacrospinous ligament appears to be of minor significance in APCII but plays an important role in vertical stabilization.

Untreated symphyseal disruption may lead to chronic instability with resulting pain and disability located to the suprapubic area or inner thigh and/or lower back/buttock [66, 67]. No data are available regarding incidence and severity of pain following non-operative management.

After surgically treated B1 injuries according to Tile moderate disability and 30–40% relevant persistent pain is reported, despite a high rate of anatomical healing [68].

Therefore, traumatic symphysis diastasis should be anatomically reduced and stable fixed for effective pain control and to allow early mobility [45, 69, 70].

**Table:** 

Stable fixation of any symphyseal disruption is clearly recommended	

**Fig. 7:**
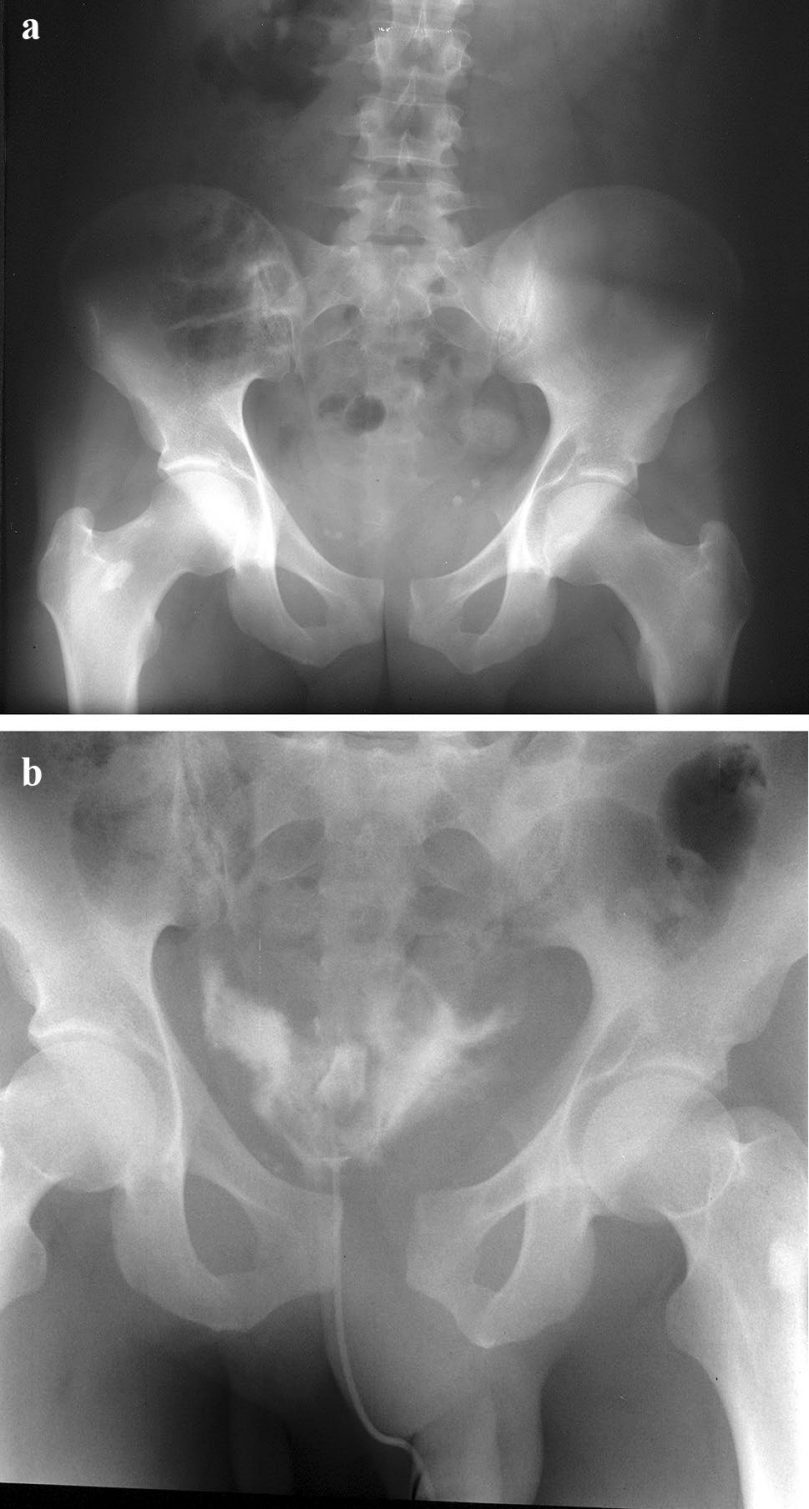
The anterior–posterior pelvic view shows symphyseal widening/separation with parallel displacement (**a**). No clear anterior SI-joint opening can be observed (**a**). External rotation stress views show a significant more displaced symphyseal disruption (**b**)

Stress radiographs and examination under anesthesia (EUA) may show the true displacement of symphyseal disruption/Instability (Fig. 7).

Suzuki et al. could show that in 6 of 22 patients (27.3%) with a symphyseal diastasis of 1–2.5 cm on conventional X-rays and an average diastasis of 1.4 cm on initial CT, demonstrated symphyseal widening > 2.5 cm during stress examination. This results in a classification change from APC-I to APC-II with additional treatment change from nonoperative to operative [71].

Sagi et al. analyzed 37 patients with APC injuries (type B according to Tile) and reported that half of the 14 patients with a suspected APC-I injuries showed symphyseal widening > 2.5 cm under stress radiography. APC-II injuries in 23 patients were further sub-classified to APC-2a (only anterior fixation) and APC-2b (combined anterior + posterior fixation: supplemental iliosacral screw) due to more severe posterior instability [72]. No correlation was reported between amount of “true” symphyseal diastasis and posterior SI-joint opening under EUA.

Grewal et al. presented another case with underestimation of plain X-ray symphyseal separation. EUA lead to significant anterior and posterior instability, indicating relevant instability, requiring surgical stabilization [73].

It is of note, that conventional x-rays overestimate diastasis by up to 50% compared to CT scans [74].

This was confirmed by Gibson et al., who analyzed 72 patients, which showed in 97% some reduction of their symphyseal separation between conventional X-ray of the pelvis and CT scan examination (26.3 vs 19.7 mm) [75].

Overall, there is a discrepancy between CT evaluation and conventional a.p. X-ray of the pelvis indicating an underestimation using CT scans alone [74, 75].

**Table:** 

Conventional x rays can mask the true displacement and therefore the amount of instability of a symphyseal disruption, when only considering horizontal displacement	

## Peripartum symphyseal instability

Peripartum diastasis of the pubic symphysis is defined as a traumatic disruption including ligamentous structures during birth due to irreversible overstretching of the lower birth canal when the newborn passes through [66, 76].

Anatomically, hormonal influence by progesterone and relaxin leads to loosening of the pelvic support structures, especially in the first trimester and during birth [42, 77], resulting in a physiologic 3–5 mm radiographic widening, which is usually corrected during the next 5 months through strengthening of the pelvic floor muscles [78, 79].

Symphyseal widening can also combined with additional posterior ring widening at the SI-joints [80].

Conservative treatment often leads to a relevant reduction of the diastasis, but residual sacroiliac symptoms may be present, especially if additional widening was present [54, 81–83].

Najibi et al. recommended to distinguish between symphyseal ligament relaxation and labor-induced symphyseal rupture (complete symphyseal disruption) [82].

**Table:** 

Based on experience with peripartum symphyseal diastasis, a non traumatic “overstretching” can lead to full ligamentous symphyseal disruption	

## Conclusion

The present biomechanical, experimental and clinical knowledge of symphyseal disruption does not allow distinguishing instability based on the well-known 2.5 cm displacement.

Independent on the amount of symphyseal displacement on standard x-rays and CT-scans, small displacement (5–10 mm) should be analyzed by stress examination, while all other disruptions with bigger displacement (widening > 10 mm) should be treated surgically.

## Data Availability

No datasets were generated or analysed during the current study.
